# Targeted metabolomic analysis of serum amino acids in heart failure patients

**DOI:** 10.1007/s00726-024-03385-7

**Published:** 2024-03-14

**Authors:** Chunjing Yang, Zhengyuan Shi, Li Bao, Xiqiao Xv, Dechun Jiang, Longtai You

**Affiliations:** 1 Department of Pharmacy, Beijing Shijitan Hospital, Capital Medical University, Beijing, 100038 China; 2 Beijing Key Laboratory of Evaluation of Rational Drug Use, Beijing, 100038 China; 3 National Center for Children’s Health, Beijing Children’s Hospital, Capital Medical University, Beijing, 100045 China

**Keywords:** Heart failure, Biomarker, Amino acids, Serum, Targeted metabolomics

## Abstract

Heart failure (HF) has been recognized as a global epidemic with high rates of morbidity, hospitalization, and mortality. The role of amino acids, which provide the body with energy, in the development of HF is still unclear. The aim of this study was to explore changes in serum amino acids in patients with HF and identify potential biomarkers. First, the serum amino acid metabolism profiles of 44 patients with HF and 30 healthy controls (Con) were quantitatively measured. Then, candidate markers were identified through the utilization of *T* test, multivariate statistical analysis, and receiver operating characteristic (ROC) curve analysis. The results found that there were 11 amino acid levels that were significantly different between patients with HF and Con. Based on ROC curve analysis, the biomarkers of eight amino acids (Glutamic acid, Taurine, l-aspartic acid, l-ornithine, Ethanolamine, l-Serine, l-Sarcosine, and Cysteine) showed high sensitivity and specificity (AUC > 0.90), and binary logistic regression analysis was used in MetaboAnalyst 5.0. Among the amino acids examined, six exhibited notable alterations in accordance with the severity of HF. In conclusion, this study cannot only provide clinicians with an objective diagnostic approach for the early identification of HF, but also enhances comprehension of the underlying mechanisms involved in the pathogenesis of HF.

**Supplementary Information:**

The online version contains supplementary material available at 10.1007/s00726-024-03385-7.

## Introduction

Heart failure (HF) is a serious condition characterized by fluid retention, dyspnea, and malaise, caused by the inability of cardiac output to meet the metabolic demands of the body's tissues due to damage to the structure or function of the heart, which results in stagnation of the pulmonary and/or somatic circulations as well as reduced perfusion of the organs (Heidenreich et al. 2022). Globally, cardiovascular diseases still lead the mortality rate, higher than tumors and other diseases. Among them, HF is one of the causes of morbidity and mortality, and its incidence continues to rise as human life increases, making it a chronic disease that jeopardizes public health and economic interests (Bozkurt et al. 2021). To date, echocardiography and NT-proBNP are regarded as the "gold standards" for the diagnosis of HF (Feng et al. 2023; Ward et al. 2022). However, echocardiography is characterized by low sensitivity and specificity, long diagnostic time, and technical complexity (Yan et al. 2018), while NT-proBNP is affected by factors such as renal failure (Takase and Dohi 2014; Bachmann et al. 2021), obesity (Bachmann et al. 2021; Parcha et al. 2021), age (Bachmann et al. 2021; Hess et al. 2005; Braisch et al. 2022), and gender (Hess et al. 2005; Braisch et al. 2022; Daubert et al. 2021), which limits its predictive accuracy. Therefore, there is an urgent need to discover new and effective diagnostic and prognostic biomarkers for HF.

Numerous studies have shown that a failing heart is like a "fuel depleted engine", unable to properly use fuel to meet the needs of metabolism (Murashige et al. 2022). To date, most cardiometabolic studies have focused on glucose, fatty acids, and lactic acid, but a growing number of studies have noted alterations in amino acids (Chen et al. 2020; Hakuno et al. 2015). The prognostic value of the branched-chain amino acids (BCAAs)/total amino acid ratio or BCAA/Aromatic amino acids (AAAs) ratio in patients with HF has been reported (Hiraiwa et al. 2020; Kimura et al. 2020). Consequently, we opted to investigate the metabolism of amino acids to identify biomarker patterns that may contribute to an enhanced comprehension of the pathophysiology of HF or facilitate early detection.

The aim of this study was to investigate amino acid metabolism in patients with HF and to analyze the relationship between changes in amino acid metabolism and disease, thereby improving our understanding of the pathogenesis of HF. Consequently, we employed liquid chromatography mass spectrometry (LC–MS/MS) to quantitatively measure the levels of 33 amino acids in the serum of HF patients and healthy controls, aiming to identify significant variations in amino acid profiles among different groups. Our findings help us better understand the development and progression of HF.

## Materials and methods

### Participants

This study was approved by the Ethics Committee of Beijing Shijitan Hospital Affiliated to Capital Medical University (sjtkyll-1x-2021(21)) in accordance with the 1964 Declaration of Helsinki and its subsequent amendments. Written informed consent was obtained from all participants. Both patients with HF (*n* = 44) and healthy controls (Con, *n* = 30) were from the population who came to Beijing Shijitan Hospital affiliated to Capital Medical University during 2021–2023. Serum samples were collected and placed in a coagulant tube, centrifuged (3000 r/min) for 10 min, then supernatant was taken, divided and stored in a refrigerator at – 80 ℃ for later use.

The inclusion criteria for patients with HF were as follows: (1) aged 20–80 years; (2) diagnosed with HF in our hospital according to Framingham criteria; (3) does not have any other clinically diagnosed serious medical condition, including but not limited to tumor, neurological, digestive or mental disorders; or infectious diseases. The sex and age of the healthy control group were matched with the patients with HF, and the level of statistical significance was > 0.05. The inclusion criteria for healthy controls were as follows: (1) aged between 20 and 80 years; (2) no history of self-reported or clinical diagnosis of any chronic serious illness within 3 months, including but not limited to tumor, neurological, kidney, digestive or mental disorders, and infectious diseases; (3) did not receive any treatment within 3 months prior to blood collection.

### Quantitative analysis of amino acids

We used the same amino acid detection protocol as in a previously published study (Shi et al. 2023). Briefly, 10 μL of serum sample (or amino acid standard) was added to 10 μL of water and 5 μL of internal standard, mixed appropriately, isopropanol was added to precipitate the protein. The mixture was centrifuged at 12,000 r/min for 10 min. The supernatant (10 μL) was then mixed with 70 μL of borate buffer and 20 μL of AccQ-Tag reagent. After vortexing for 10 s, the mixture was heated at 55 °C for 10 min. We detected amino acids in the samples using UPLC-MS/MS in positive ion multiple reaction monitoring (MRM) mode on a Xevo TQ-XS time-of-flight mass spectrometer (Waters, Shanghai, China). Amino acid abbreviations and mass spectrometric conditions for amino acids and isotopic internal standards are listed in Supplementary Table S1 and S2. Supplementary Table S3 lists the gradient procedure for liquid chromatography.

### Statistical analysis

*T* tests or Wilcoxon tests were performed with SPSS 19.0 statistical software to compare the mean differences between two groups. When conducting multiple tests, apply False Discovery Rate (FDR) adjustment as needed to correct for multiple comparisons. Next, the web-based metabolomics software MetaboAnalyst 5.0 (https://www.metaboanalyst.ca/) was used for analysis. The differentiation between the HF and Con groups was achieved through the utilization of unsupervised Principal Component Analysis (PCA) and Orthogonal Partial Least Squares Discriminant Analysis (OPLS-DA). The variable importance (VIP) was computed for each amino acid projection in the model to demonstrate its contribution to the classification task. Statistical significance was determined by assessing variables with a VIP value exceeding 1.0 and a p value below 0.05. We also performed heat map and receiver operating characteristic curve (ROC) analyses.

## Results

### Baseline characteristics

The HF patients were classified according to NYHA, including 5 cases in grade II, 17 cases in grade III, and 22 cases in grade IV. In addition, we compared different subgroups of HF patients with the control group. Details of clinical features are presented in Table 1. The box plots of clinical features with significant digits are provided in Supplementary Fig. S1.

**Table 1:** Baseline characteristics of participants

Clinical information	HF (*n* = 44)			Con (*n* = 30)
	HF-II	HF-III	HF-IV	
Age, year	66.5 ± 8.8	66.5 ± 10.0	66.7 ± 11.1	66.44 ± 11.47
Sex, *n*	Male (3)	Male (7)	Male (10)	Male (16)
Smoking, *n* (%)	2 (40)	10 (59)	15(68)	10 (33)
Drinking, *n* (%)	3(60)	5(29)	3 (11)	17 (57)
NYHA Class, *n* (percentage of 44)				
II	5 (11%)	0 (0%)	0 (0%)	0 (0%)
III	0 (0%)	17 (39%)	0 (0%)	0 (0%)
IV	0 (0%)	0 (0%)	22 (50%)	0 (0%)
NT-proBNP (pg/mL)	4115.8 ± 1454.0^***^	4368.8 ± 2140.8^***^	5610.0 ± 1579.0^***^	517.17 ± 207.01
Atrial fibrillation, *n*	0	1	5	0
Ventricular tachycardia, *n*	0	2	1	0
Plasma D-dimer (ng/mL)	3910.6 ± 3972.6^***^	3594.0 ± 2174.1^***^	3012.4 ± 1895.7^***^	496.41 ± 213.87
CRP (mg/L)	75.1 ± 20.4^***^	95.4 ± 44.8^***^	98.5 ± 32.3^***^	14.57 ± 11.44
Creatinine (μM)	152.3 ± 155.5^*^	239.1 ± 148.9^***^	256.1 ± 227.8^***^	77.70 ± 33.89
Albumin (g/L)	30.6 ± 1.8	28.6 ± 5.1	29.1 ± 2.0	34.17 ± 3.19
Cardiac troponin I (pg/mL)	29.3 ± 22.7^*^	74.0 ± 73.3	61.7 ± 76.1	56.63 ± 32.60
Creatine kinase-MB isoenzyme mass (ng/mL)	2.0 ± 1.7	3.6 ± 2.8^***^	2.9 ± 2.9^**^	1.00 ± 1.44

*HF* heart failure, *Con* control, *n* number of patients, *NYHA Class* New York Heart Association Functional Classification, *NT-proBNP* N-terminal pro-brain natriuretic peptide, *CRP* C-reactive protein, *p < 0.05, **p < 0.01, ***p < 0.001 vs control group.

### MRM chromatogram of amino acid standards

**Fig. 1:**
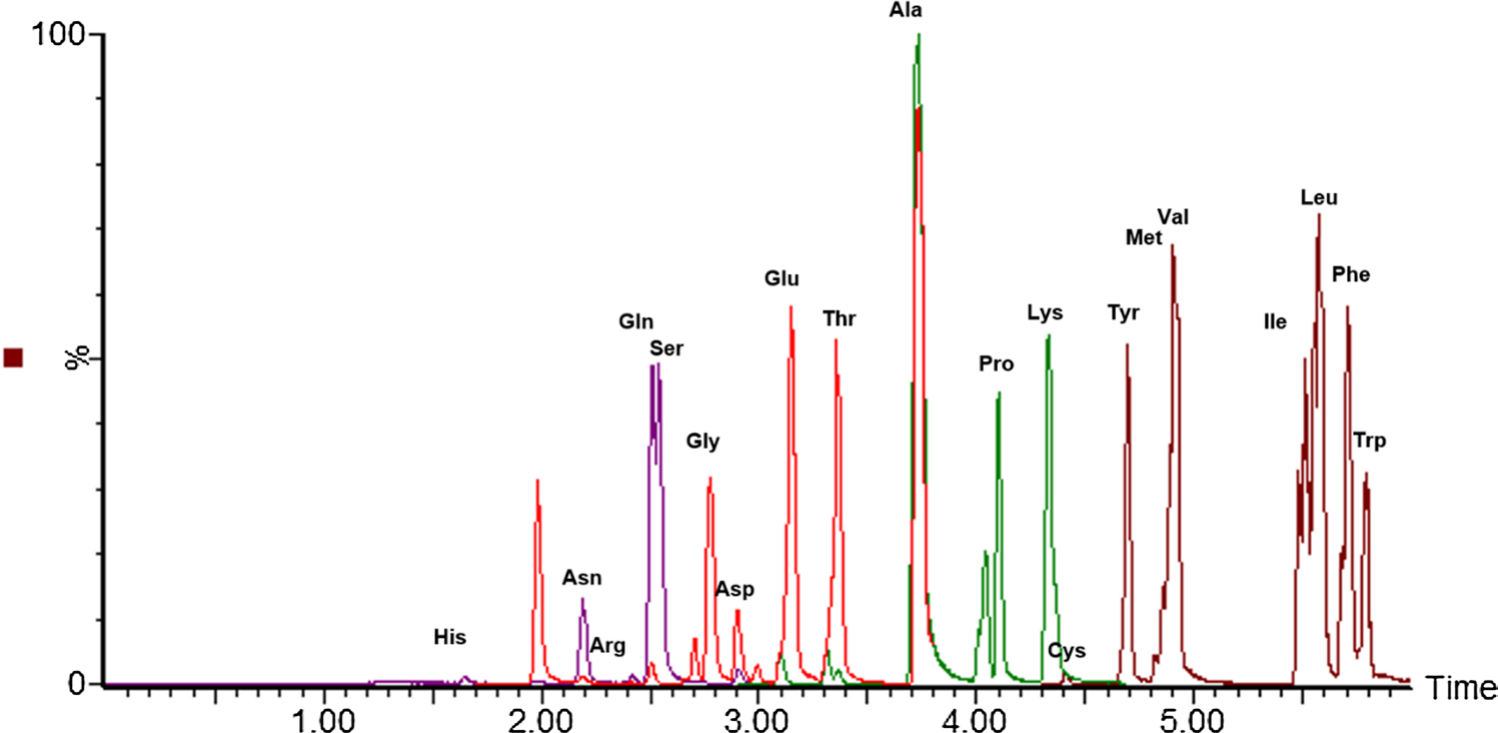
A representative MRM chromatogram of amino acid quantification

We measured 33 amino acids, but due to overlapping peaks, we only labeled 20 standard amino acids that constitute proteins in Fig. 1.

### Principal component analysis

**Fig. 2:**
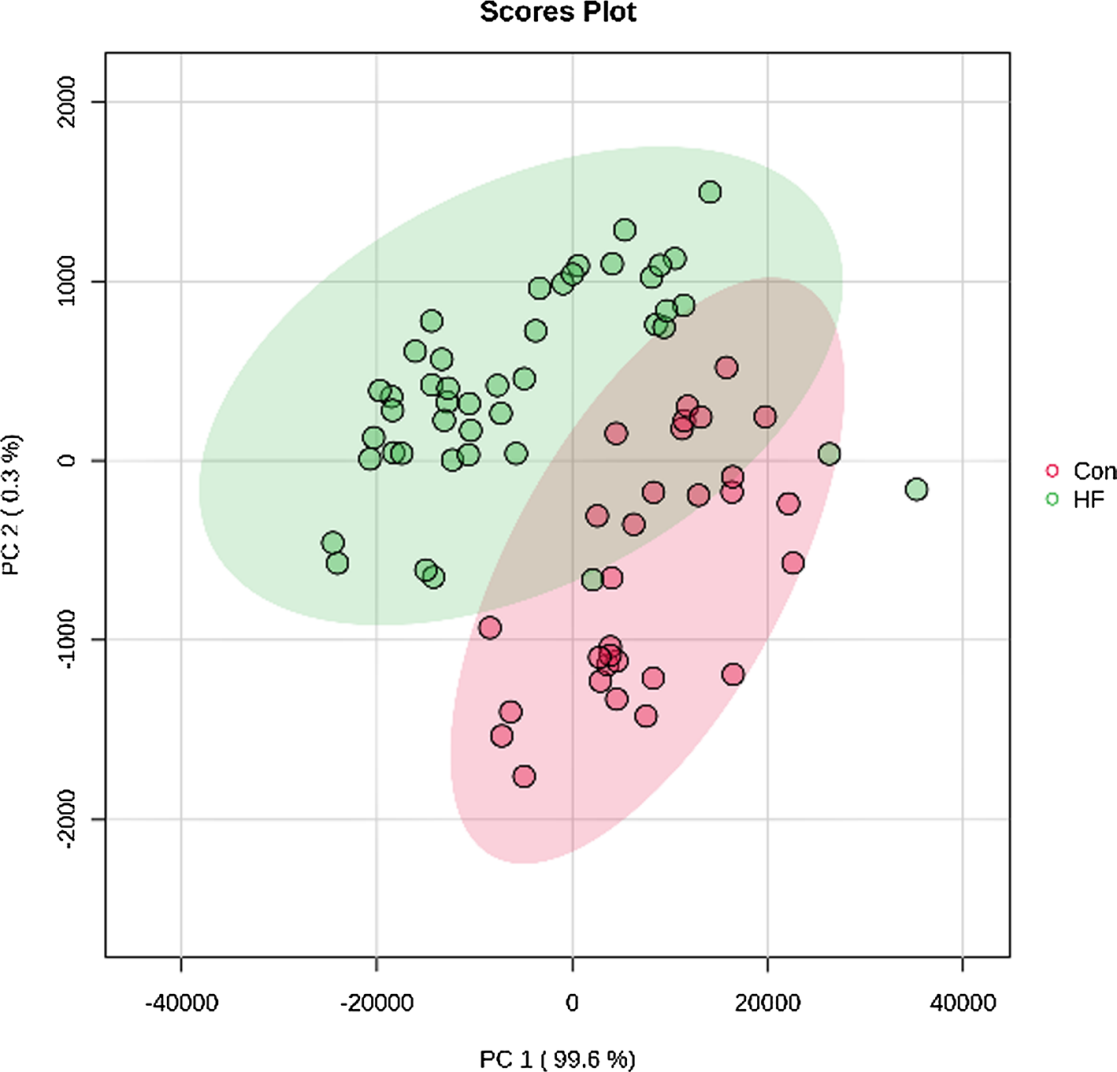
PCA scores plots of HF and control (Con) group

Principal component analysis (PCA) is used to study the natural distribution of amino acids. Figure 2 shows the PCA plots of the HF group and control group. It can be seen from the PCA diagram that the serum samples are distributed in different regions under different physiological states, indicating that there are differences in the composition and concentration of amino acids. Because clinical samples are affected by many factors, these factors inevitably produce a large number of noisy signals unrelated to information grouping. Therefore, there is a partial overlap in the PCA score plot that cannot be explained. PCA with QC is shown in Supplementary Fig. S2. Standard curve linearity, matrix effects and QCs obtained for the analytes are provided in Supplementary Tables S4 and S5.

### Screening and identification of differential amino acids as potential biomarkers of HF

#### Hierarchical cluster analysis

**Fig. 3:**
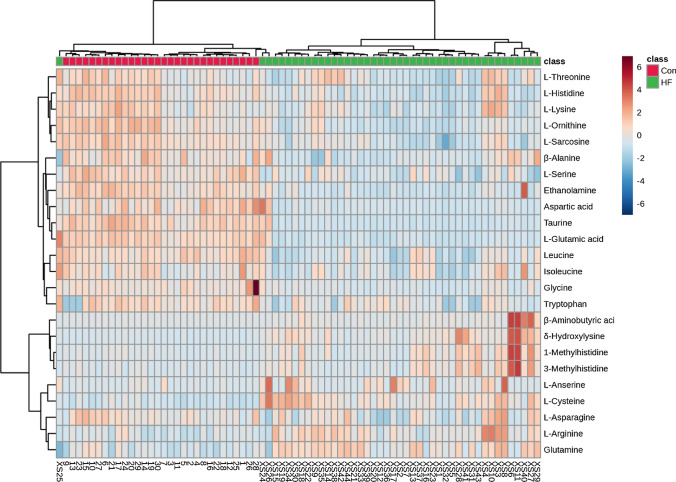
Heat map of differential amino acids in HF and controls (Con). The color of each section is proportional to the significance of the amino acid change (red, upregulated; blue, downregulated). Rows, samples; columns, amino acids

Heat maps visually represent differences in serum amino acids between patients with HF and controls. To better understand the metabolic changes of the two groups of amino acids, we performed hierarchical cluster analysis on amino acid concentration data obtained from targeted LC–MS analysis. Although some samples overlapped slightly, most samples were clearly divided into two distinct clusters, which is consistent with PCA analysis (Fig. 3).

#### Orthogonal partial least squares discriminant analysis

**Fig. 4:**
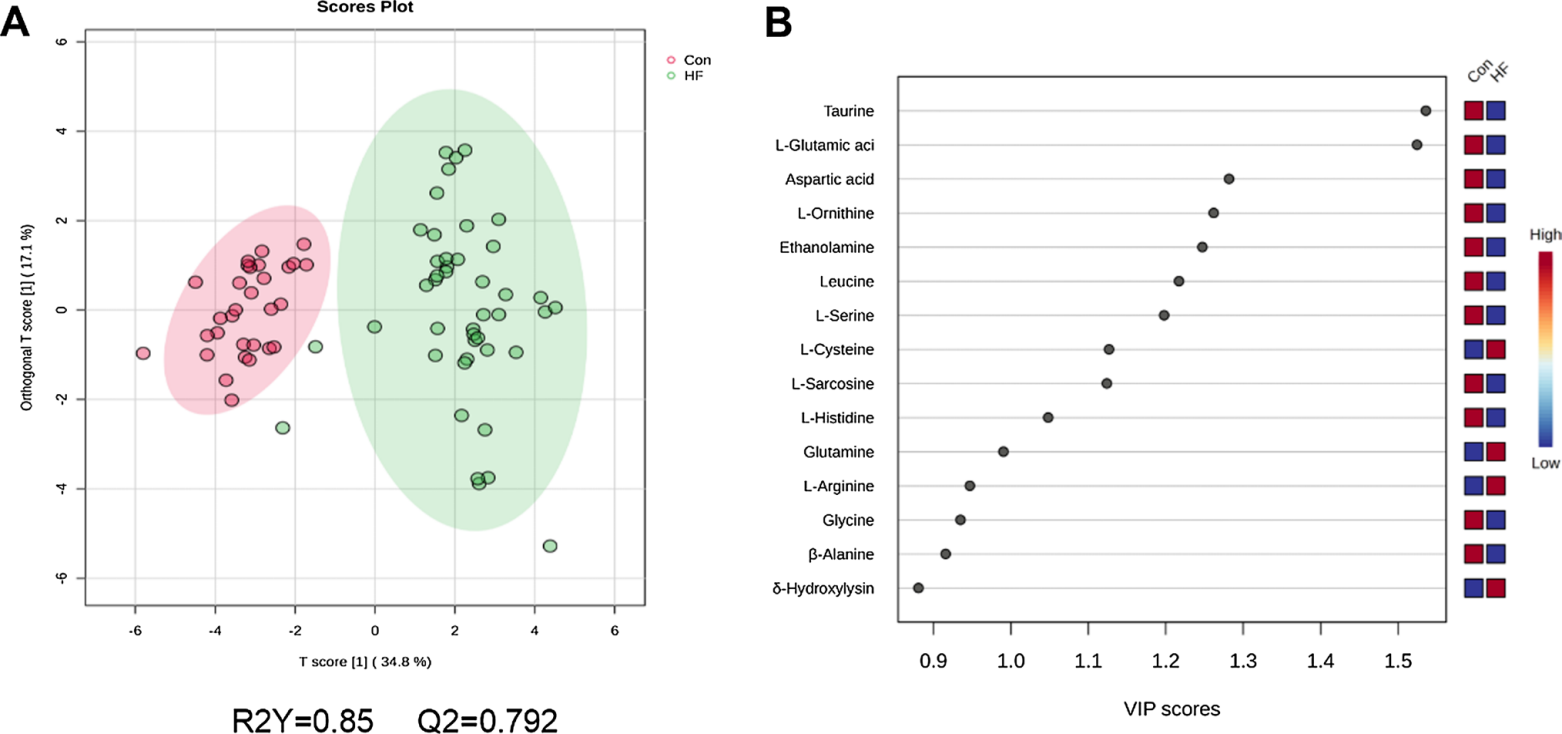
OPLS-DA analysis of HF and control (Con) group. **A** OPLS-DA scores plots; **B** Top 15 amino acids for VIP score. VIP scores represent the importance of the amino acids involved in the separation in the OPLS-DA model; R2Y represent the percentage of response variance explained by the full model; Q2 represents the predictive performance of the model estimated by cross validation

Orthogonal partial least squares discriminant analysis (OPLS-DA, Fig. 4A) models showed a clear separation trend between the two groups, revealing more clearly the differences between HF and control. R2Y represents the explanatory rate of the model to the Y matrix, and Q2 represents the predictive power of the model. Our results show that R2Y = 0.85, Q2 = 0.79, both of which are close to 1, indicating that the model is good and the fitting accuracy of the model is high. Figure 4B shows that there are 11 amino acids with VIP scores > 1.0 obtained by the OPLS-DA model. The colored boxes on the right represent the relative concentrations of the corresponding amino acids in the two groups.

#### Classification model to distinguish HF and control

**Fig. 5:**
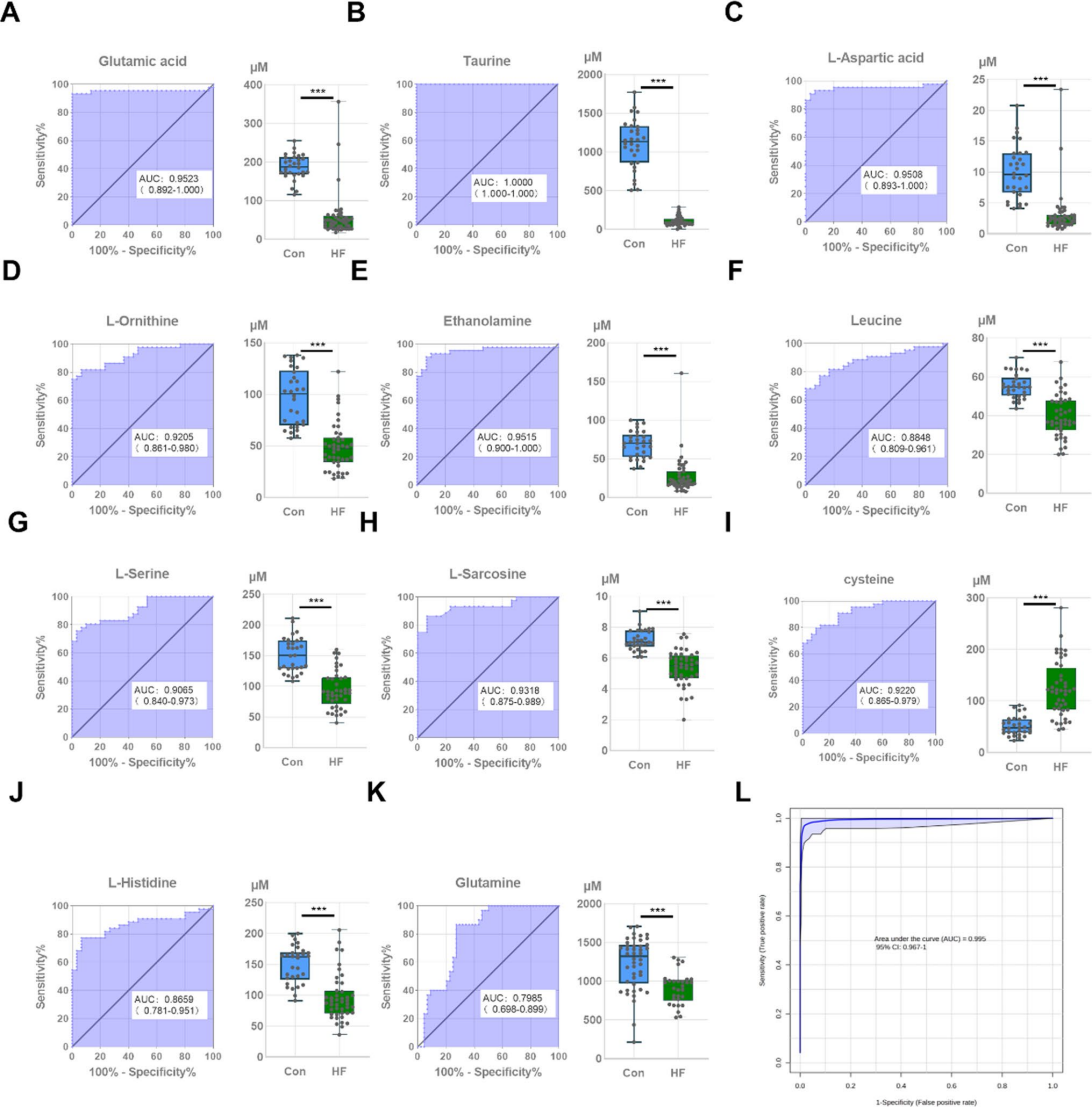
The diagnostic value of differential amino acids was determined. Subject work characterization (ROC) analyses revealed the diagnostic performance of these differential amino acids. AUC (0.5–0.7), low accuracy; AUC (0.7–0.9), moderate accuracy; AUC (> 0.9), high accuracy

The potential of the 11 amino acids screened before as diagnostic biomarkers for HF was evaluated by ROC curve analysis (Fig. 5). The sensitivity values and area under the ROC curve (AUC) of these amino acids were > 0.7 (Fig. 5A–K). The AUC value close to 1 indicates higher accuracy. Of these, eight amino acids showed high effectiveness (AUC > 0.90) and the other three amino acids showed moderate effectiveness (AUC = 0.7–0.9). In addition, MetaboAnalyst 5.0 was used to perform binary logistic regression for the top eight amino acids (Glutamic acid, Taurine, L-Aspartic acid, L-Ornithine, Ethanolamine, L-Serine, L-Sarcosine, and Cysteine) with the best sensitivity and specificity (AUC = 0.995; Fig. 5L). These results suggest that these eight amino acids could serve as potential biomarkers for early diagnosis or treatment of HF.

### Amino acid levels in HF patients stratified by disease severity

To provide valuable insights into the potential variations in amino acid profiles associated with different stages of the disease, the HF patient cohort was divided into three groups based on disease severity for a more detailed analysis of amino acid levels in relation to the severity of HF. Table 2 demonstrates the amino acid levels of the different groups. Notably, six amino acids (Taurine, l-Serine, Ethanolamine, Aspartic acid, Glutamic acid, and Cysteine) demonstrated substantial variations across the severity groups. These findings suggest a potential correlation between disease severity and alterations in amino acid metabolism in HF patients.

**Table 2:** List of amino acid concentrations with significant digits between HF and controls (Con) group

AAs (μM)	HF (total)	HF-II	HF-III	HF-IV	Con
l-Histidine	95.995 ± 37.500^***^	123.405 ± 21.138	108.950 ± 46.139^***^	79.756 ± 23.667^***^	149.978 ± 30.116
1-Methylhistidine	11.062 ± 10.845^***^	3.213 ± 1.125	8.747 ± 5.857^***^	14.634 ± 13.492^***^	4.070 ± 1.474
3-Methylhistidine	12.528 ± 12.206^***^	4.448 ± 1.495	9.979 ± 7.170^***^	16.334 ± 15.146^***^	4.361 ± 2.520
l-Asparagine	56.188 ± 22.063^*^	54.743 ± 22.769	56.437 ± 27.955	56.325 ± 16.654^*^	65.381 ± 13.654
l-Arginine	51.840 ± 35.041^***^	51.262 ± 27.013^**^	57.159 ± 45.960^***^	47.862 ± 26.296^***^	21.650 ± 15.705
l-Anserine	0.612 ± 0.751^**^	0.590 ± 0.525^**^	0.894 ± 0.814^***^	0.425 ± 0.708	0.156 ± 0.170
Taurine	105.332 ± 59.466^***^	156.691 ± 65.718^***^	91.675 ± 34.260^***^	104.214 ± 67.364^***^	1104.446 ± 318.688
Glutamine	980.980 ± 269.336^***^	860.473 ± 401.761^***^	983.825 ± 246.826^***^	1006.170 ± 244.051^***^	1401.023 ± 191.887
l-Serine	94.332 ± 30.807^***^	105.203 ± 32.762^**^	93.811 ± 38.008^***^	91.937 ± 22.083^***^	151.174 ± 28.624
Ethanolamine	27.305 ± 24.395^***^	36.434 ± 14.904^***^	31.728 ± 35.049^***^	21.813 ± 12.743^***^	68.631 ± 17.096
Glycine	250.888 ± 82.075^***^	296.965 ± 100.491	261.896 ± 93.557^**^	231.909 ± 61.148^***^	466.736 ± 291.088
Aspartic acid	3.148 ± 3.726^***^	3.868 ± 1.157^**^	3.712 ± 5.172^***^	2.549 ± 2.640^***^	10.063 ± 4.310
l-Glutamic acid	58.403 ± 59.347^***^	100.701 ± 128.441^**^	57.750 ± 52.192^***^	49.295 ± 26.367^***^	187.113 ± 32.363
β-Alanine	122.566 ± 49.256^***^	84.893 ± 6.362^**^	117.051 ± 32.004^***^	132.411 ± 60.716^**^	174.223 ± 43.338
l-Threonine	203.058 ± 96.221^**^	247.958 ± 99.057	208.199 ± 90.044^*^	188.880 ± 98.651^**^	256.999 ± 65.204
β-Aminobutyric acid	22.201 ± 45.885^*^	3.611 ± 2.882	7.600 ± 6.044^*^	36.873 ± 60.932^**^	2.222 ± 1.644
δ-Hydroxylysine	62.175 ± 55.225^***^	35.336 ± 26.520^***^	47.784 ± 37.960^***^	79.395 ± 65.800^***^	13.076 ± 4.460
l-Ornithine	48.786 ± 23.163^***^	70.406 ± 33.647	45.556 ± 21.635^***^	46.369 ± 18.617^***^	96.953 ± 27.311
l-Cysteine	123.512 ± 54.167^***^	106.374 ± 52.032^***^	122.982 ± 46.813^***^	127.817 ± 60.033^***^	51.325 ± 18.407
Leucine	39.618 ± 10.878^***^	45.804 ± 12.883^*^	40.882 ± 11.046^***^	37.236 ± 9.668^***^	55.286 ± 6.310
Isoleucine	81.120 ± 7.061^***^	84.260 ± 8.399^*^	82.994 ± 8.094^***^	78.959 ± 5.030^***^	84.917 ± 5.075
Tryptophan	33.011 ± 17.422^***^	53.799 ± 16.721	38.447 ± 10.233^*^	24.087 ± 16.276^***^	48.446 ± 18.566
l-Sarcosine	5.372 ± 1.134^***^	6.096 ± 1.047^**^	5.533 ± 1.186^***^	5.083 ± 1.057^***^	7.167 ± 0.664
l-Lysine	283.427 ± 113.063^***^	318.030 ± 112.091	304.046 ± 141.522^*^	259.631 ± 81.862^***^	382.770 ± 92.133
Carnosine	36.182 ± 28.180	39.091 ± 20.082	47.061 ± 38.231	27.114 ± 15.247^**^	37.431 ± 12.067
Methionine	27.195 ± 14.097	37.894 ± 19.408^***^	32.767 ± 14.376^*^	20.964 ± 8.393^*^	24.311 ± 4.710
Valine	186.548 ± 57.045	193.382 ± 106.144	205.267 ± 36.768	170.530 ± 48.858^**^	204.702 ± 23.363

*AAs* amino acids, *HF (total)* the heart failure patients in total, *HF-II* the heart failure patients in grade II according to NYHA class, *HF-III* the heart failure patients in grade III according to NYHA class, *HF-IV* the heart failure patients in grade IV according to NYHA class, *Con* control, *p < 0.05, **p < 0.01, ***p < 0.001 vs control group

## Discussion

The metabolic and regulatory functions of amino acids are known to be significant, leading to variations in amino acid levels between patients with different diseases and healthy individuals. Numerous studies have reported alterations in amino acid concentrations in relation to chronic kidney disease (Wang et al. 2019), cardiovascular disease (Li et al. 2017), coronary artery disease and type-2 diabetes mellitus (Magnusson et al. 2013). Therefore, in this study, targeted metabolomics using LC–MS/MS was performed to analyze the differential amino acids between HF patients and healthy controls. We then performed a series of analyses of these differential amino acids, including heat mapping, diagnostic value assessment, and identified several amino acids with high diagnostic value.

Glutamic acid is the most abundant amino acid in the human body and is involved in a variety of physiological functions, such as synthesizing proteins, stimulating immune responses, and regulating cell regulation (Newsholme et al. 2003). Several studies have demonstrated that glutamic acid is among the limited number of amino acids obtained from the heart shortly after coronary artery bypass grafting, and it is the most prevalent amino acid extracted from both the heart and skeletal muscle in such circumstances (Svedjeholm et al. 1991, 1990). Taurine, a semiessential amino acid, highly esteemed for its properties, is the predominant free amino acid found in cardiac myocytes and exhibits beneficial antifibrotic and anti-free radical effects (Ito et al. 2014). Taurine has been reported to reduce cardiomyocyte hypertrophy and fibrosis, and has significant therapeutic effects in chronic HF (Liu et al. 2020). It is currently approved for HF treatment in Japan (Roşca et al. 2022).

Histidine levels decreased and cystine levels increased in patients with HF, which was consistent with literature reports (Kuo et al. 2019; Tessari 2019; Hakuno et al. 2015). Histidine cannot be synthesized in the human body, but it has important physiological functions and is a dietary essential amino acid (Brosnan and Brosnan 2020). First, histidine is a precursor to the synthesis of histamine. Histamine is a bioactive substance that plays a role in regulating vasomotor and blood flow regulation in the cardiovascular system. In patients with HF, histamine may be involved in the regulation of inflammation and cardiovascular function, so histidine, as a precursor of histamine, may be related to the onset and progression of HF (Huang 1985). Second, histidine plays an important role in improving oxidative stress and nitric oxide (NO) metabolism (Yang et al. 2023; Jiang et al. 2016). Oral administration of histidine improves the metabolic pattern in serum and renal tissues of salt-sensitive hypertensive rats, decreases ROS, and increases NO content (Yang et al. 2021). NO has many physiological functions in the cardiovascular system, such as vasodilation and anti-inflammatory effect (McIntyre and Dominiczak 1997). Therefore, histidine metabolism and NO production may play a potential role in the treatment of HF. Finally, histidine has certain antioxidant properties, which can clear free radicals and oxidative stress, reduce myocardial damage and inflammatory response (Megías et al. 2007). Patients with HF are often accompanied by increased oxidative stress, and the antioxidant effect of histidine may help to protect and improve the pathological process of HF (Romuk et al. 2019). Cystine is a sulfur-containing amino acid that plays an important physiological function in human body. The study found that plasma and tissue cystine levels were significantly elevated in patients with HF (Kuo et al. 2019; Tessari 2019; Hakuno et al. 2015). Elevated cystine levels can cause damage to vascular endothelial cells and constriction of blood vessels, which may be associated with the development of HF (Xia et al. 2020).

The stratification of the HF patient cohort based on disease severity has provided valuable insights into the relationship between amino acid profiles and HF progression. Amino acids, as the basic components of protein and peptide synthesis, are essential for normal growth and development of the body. The observed variations in specific amino acids among the severity groups indicate that alterations in amino acid metabolism may be associated with the severity of HF. Inflammatory marker C-reactive protein (CRP) is a common inflammatory cytokine, which plays a key role in the occurrence and development of cardiac dysfunction. With the aggravation of HF, the level of CRP increases. It is worth noting that studies have found that higher levels of taurine are associated with lower levels of CRP, and taurine can significantly reduce the level of inflammatory cytokine CRP, thereby alleviating the damage mediated by inflammatory mediators, and thus may play a myocardial protective effect (Singh et al. 2023; Zhang et al. 2018). Clinical assessment of the severity of HF is often combined with cardiac ultrasound, cardiac MRI and patient symptoms, such as shortness of breath, edema, etc. Amino acids have not been used as indicators to assess the severity of HF, and our study has conducted a preliminary exploration in this respect. These findings warrant further investigation to better understand the underlying mechanisms and potential clinical implications.

Our study highlights plasma amino acid analysis as a potentially useful tool to explore markers for HF risk assessment and prognosis. However, we should acknowledge that the study has some limitations. Most of our results are consistent with previous studies, but there are some inconsistencies. First of all, the small sample size may reduce the statistical power, and this experiment can only be used as a preliminary study, and the accuracy of the eight different amino acids identified as potential biomarkers needs more research to verify. Second, the metabolism of amino acids in the body is relatively complex, and the changes of amino acid metabolites and related specific metabolic pathways will be focused on in the following studies. On the other hand, due to the nature of single-center case–control studies, larger studies are needed to answer other questions before the identified amino acids can be used in clinical decision-making, such as whether amino acid-directed therapy can improve treatment outcomes.

## Conclusion

In this study, the levels of 11 amino acids were differentially regulated in the serum of patients with HF, while further validation showed that 8 amino acids may be most associated with the disease state. This extends our understanding of the pathophysiology of the disease. Future studies could identify appropriate biomarkers for early detection to help physicians diagnose HF early and guide treatment and prognosis.

## Supplementary Information

Below is the link to the electronic supplementary material.

Supplementary file1 (DOCX 427 KB)

## Data Availability

Data sets used and/or analyzed in this study are available on request from the corresponding authors.
