# Supplementary material for: Targeted metabolomic analysis of serum amino acids in heart failure patients
Source: Amino Acids. 2024 Mar 14;56:22. doi: 10.1007/s00726-024-03385-7 (PMC10940394; doi:10.1007/s00726-024-03385-7)
Supplement: Supplementary file 1 [file 726_2024_3385_MOESM1_ESM.docx]

Supplementary Material

**Targeted metabolomic analysis of serum amino acids in heart failure patients**

**Chunjing Yang^1,2^ · Zhengyuan Shi^1,2^ · Li Bao^1,2^ · Xiqiao Xv^1,2^ · Dechun Jiang^1,2^ · Longtai You^3^**

**Supplementary Table 1 Amino Acid Abbreviations**

|  | **Abbreviations** | **Amino Acid** |
| --- | --- | --- |
| 1 | 1-MEHIS | 1-Methylhistidine |
| 2 | 3-MEHIS | 3-Methylhistidine |
| 3 | ALA | Alanine |
| 4 | AMADP | α-Aminoadipic acid |
| 5 | ANS | Anserine |
| 6 | ARG | Arginine |
| 7 | ASN | Asparagine |
| 8 | ASP | Aspartic acid |
| 9 | BETA-GABA | β-Aminobutyric acid |
| 10 | CAR | carnosine |
| 11 | CIT | Citrulline |
| 12 | CYS | cysteine |
| 13 | ETH | Ethanolamine |
| 14 | γ-GABA | γ-Aminobutyric acid |
| 15 | GLN | Glutamine |
| 16 | GLU | Glutamic acid |
| 17 | GLY | Glycine |
| 18 | HIS | Histidine |
| 19 | ILE | Isoleucine |
| 20 | LEU | Leucine |
| 21 | LYS | Lysine |
| 22 | MET | Methionine |
| 23 | OH-LYS | δ-Hydroxylysine |
| 24 | ORN | Ornithine |
| 25 | PHE | Phenylalanine |
| 26 | PRO | Proline |
| 27 | SAR | Sarcosine |
| 28 | SER | Serine |
| 29 | TAU | Taurine |
| 30 | THR | Threonine |
| 31 | TRP | Tryptophan |
| 32 | TYR | Tyrosine |
| 33 | VAL | Valine |

**Supplementary Table 2 Mass Spectrometric Conditions**

| **Compound** | **Parent ion (m/z)** | **Daughter ion (m/z)** | | **Cone(V)** | **Collision(V)** | | **Dwell（s）** |
| --- | --- | --- | --- | --- | --- | --- | --- |
| ETH | 232.1 | 171.1 | 10 | | | 20 | 0.003 |
| GLY | 246.1 | 116.1 | 30 | | | 40 | 0.003 |
| GLY-d2 | 248.1 | 171.1 | 30 | | | 20 | 0.003 |
| ALA | 260.1 | 116.1 | 30 | | | 44 | 0.003 |
| SAR | 260.1 | 171.1 | 30 | | | 20 | 0.003 |
| THR | 290.1 | 171.1 | 30 | | | 20 | 0.003 |
| THR-d5 | 295.1 | 171.1 | 30 | | | 26 | 0.003 |
| ASP | 304.1 | 171.1 | 30 | | | 22 | 0.003 |
| ASP-d3 | 307.1 | 171.1 | 30 | | | 22 | 0.003 |
| GLU | 318.1 | 171.1 | 30 | | | 22 | 0.003 |
| GLU-d5 | 323.1 | 171.1 | 30 | | | 22 | 0.003 |
| CIT | 346.2 | 171.1 | 30 | | | 26 | 0.003 |
| ORN | 237.0 | 171.1 | 30 | | | 20 | 0.003 |
| LYS | 244.1 | 171.1 | 30 | | | 12 | 0.003 |
| LYS-d4 | 248.1 | 171.1 | 30 | | | 12 | 0.003 |
| ALA-d4 | 264.1 | 171.1 | 30 | | | 20 | 0.003 |
| β-GABA | 274.1 | 171.1 | 10 | | | 20 | 0.003 |
| γ-GABA | 274.1 | 171.1 | 10 | | | 20 | 0.003 |
| PRO | 286.1 | 116.1 | 30 | | | 50 | 0.003 |
| CYS | 291.1 | 171.1 | 10 | | | 12 | 0.003 |
| PRO-d7 | 293.1 | 171.1 | 30 | | | 24 | 0.003 |
| AMADP | 332.1 | 171.1 | 30 | | | 18 | 0.003 |
| OH-LYS | 333.2 | 171.1 | 16 | | | 16 | 0.003 |

**(continued)**

**Supplementary Table 2**

**(continued)**

| **Compound** | **Parent ion (m/z)** | **Daughter ion (m/z)** | | **Cone(V)** | **Collision(V)** | | **Dwell（s）** |
| --- | --- | --- | --- | --- | --- | --- | --- |
| SER | 276.1 | 171.1 | 30 | | | 20 | 0.003 |
| SER-d3 | 279.1 | 171.1 | 30 | | | 20 | 0.003 |
| TAU | 296.1 | 116.3 | 30 | | | 60 | 0.003 |
| ASN | 303.1 | 171.1 | 30 | | | 22 | 0.003 |
| ASN-d3 | 306.1 | 171.1 | 30 | | | 22 | 0.003 |
| GLN | 317.1 | 171.1 | 30 | | | 24 | 0.003 |
| GLN-d5 | 322.1 | 171.1 | 29 | | | 24 | 0.003 |
| HIS | 326.1 | 156.1 | 20 | | | 10 | 0.003 |
| HIS-d3 | 329.1 | 159.1 | 20 | | | 10 | 0.003 |
| 1-MEHIS | 340.1 | 124.2 | 30 | | | 28 | 0.003 |
| 3-MEHIS | 340.1 | 170.1 | 30 | | | 18 | 0.003 |
| ARG | 345.1 | 70.1 | 30 | | | 36 | 0.003 |
| ARG-d7 | 352.1 | 70.1 | 31 | | | 36 | 0.003 |
| CAR | 397.1 | 227.2 | 30 | | | 14 | 0.003 |
| ANS | 411.1 | 241.1 | 68 | | | 18 | 0.003 |
| VAL | 288.1 | 171.1 | 30 | | | 16 | 0.003 |
| VAL-d8 | 296.1 | 171.1 | 30 | | | 20 | 0.003 |
| ILE | 302.1 | 171.1 | 30 | | | 20 | 0.003 |
| ILE-d10 | 312.1 | 171.1 | 30 | | | 20 | 0.003 |
| LEU | 302.1 | 171.1 | 30 | | | 20 | 0.003 |
| LEU-d10 | 312.1 | 171.1 | 30 | | | 20 | 0.003 |
| TRP | 375.1 | 171.1 | 30 | | | 26 | 0.003 |

**(continued)**

**Supplementary Table 2**

**(continued)**

| **Compound** | **Parent ion (m/z)** | **Daughter ion (m/z)** | | **Cone(V)** | **Collision(V)** | | **Dwell（s）** |
| --- | --- | --- | --- | --- | --- | --- | --- |
| TRP-d8 | 383.1 | 171.1 | | 30 | 26 | | 0.003 |
| TYR | 352.1 | 171.1 | 30 | | | 24 | 0.003 |
| TYR-d7 | 359.1 | 171.1 | 30 | | | 24 | 0.003 |
| MET | 320.1 | 171.1 | 30 | | | 22 | 0.003 |
| PHE | 336.1 | 171.1 | 30 | | | 22 | 0.003 |
| PHE-d8 | 344.1 | 116.3 | 30 | | | 22 | 0.003 |

**Supplementary Table 3 The gradient procedure for liquid chromatography**

| **Time (min)** | **Flow (mL/min)** | **A（%）** | **B (%)** | **CURVE** |
| --- | --- | --- | --- | --- |
| 0.0 | 0.5 | 4 | 96 |  |
| 0.5 | 0.5 | 4 | 96 | 6 |
| 2.5 | 0.5 | 10 | 90 | 6 |
| 5.0 | 0.5 | 28 | 72 | 6 |
| 5.1 | 0.5 | 95 | 5 | 6 |
| 6.1 | 0.5 | 95 | 5 | 6 |
| 6.2 | 0.5 | 4 | 96 | 6 |
| 7.5 | 0.5 | 4 | 96 | 6 |

A: Acetonitrile containing 0.1% formic acid (v/v); B: Water containing 0.1% formic acid (v/v)

ACQUITY UPLC HSS T3 column (150 mm × 2.1 mm, 1.8 μm);

Column temperature: 55 ℃;

Injector temperature: 4 ℃;

Injection volume: 2 μL.

**Table S4 Standard curve linearity and QC data obtained for the analytes**

| **Compound** | **Linear equation** | **r^2^** | **Linearity range（μM）** | **QCL** | | **QCM** | | **QCH** | |
| --- | --- | --- | --- | --- | --- | --- | --- | --- | --- |
|  |  |  |  | **RE/%** | **RSD/%** | **RE/%** | **RSD/%** | **RE/%** | **RSD/%** |
| HIS^*^ | Y=435.671X-203.442 | 0.991062 | 1~400 | 3.8 | 1.6 | 3.9 | 1.5 | 4.5 | 1.3 |
| 1-MEHIS^*^ | Y=280.869X+38.3116 | 0.992624 | 1~200 | 1.3 | 3.7 | 1.4 | 3.4 | 2.0 | 2.4 |
| 3-MEHIS^*^ | Y=214.998X-65.7157 | 0.981024 | 1~200 | -1.9 | -2.0 | -1.7 | -2.2 | -1.1 | -3.3 |
| ASN^*^ | Y= 0.0211011X+0.0183646 | 0.981569 | 2~200 | 2.3 | 2.0 | 2.5 | 1.9 | 3.1 | 1.5 |
| ARG^*^ | Y=214.074X+124.486 | 0.990419 | 2~400 | 4.3 | 3.0 | 4.4 | 2.9 | 5.0 | 2.6 |
| ANS^*^ | Y=497.554X-18.9354 | 0.989184 | 1~400 | 5.3 | 3.8 | 5.5 | 3.7 | 6.1 | 3.4 |
| TAU^#^ | Y=47.8266X-36.8685 | 0.991327 | 50~1500 | 5.9 | 0.5 | 6.0 | 0.5 | 6.6 | 0.5 |
| GLN^#^ | Y=0.0132278X+16.0512 | 0.987876 | 50~1500 | 4.6 | 2.4 | 4.8 | 2.3 | 5.4 | 2.1 |
| SER^*^ | Y=0.0327862X+0.049597 | 0.993259 | 2~200 | 2.4 | 9.2 | 2.6 | 8.7 | 3.2 | 7.1 |
| ETH^*^ | Y=4752.47X+4621.32 | 0.997601 | 1~200 | 2.4 | 9.2 | 2.6 | 8.7 | 3.2 | 7.1 |
| GLY^#^ | Y=0.0022196X+0.136896 | 0.990429 | 50~1500 | 1.6 | 1.4 | 1.8 | 1.3 | 2.4 | 1.0 |
| ASP^*^ | Y=0.0687181X+0.027017 | 0.994847 | 1~200 | 2.7 | 0.8 | 2.8 | 0.7 | 3.4 | 0.6 |
| GLU^*^ | Y=0.0158414X+0.0038762 | 0.992655 | 2~400 | 6.3 | 1.4 | 6.4 | 1.4 | 7.0 | 1.3 |
| ALA^*^ | Y=1770.01X+10909.1 | 0.995357 | 2~400 | 8.1 | 2.3 | 8.2 | 2.2 | 8.8 | 2.1 |
| THR^*^ | Y=13483.1X+8468.73 | 0.994037 | 2~400 | 0.6 | 1.0 | 0.7 | 0.7 | 1.3 | 0.4 |
| β-GABA^*^ | Y=20972.6X+7144.86 | 0.987773 | 1~200 | 2.1 | 3.5 | 2.2 | 3.3 | 2.8 | 2.6 |
| OH-LYS^*^ | Y=82.1403X+32.1412 | 0.994687 | 2~200 | 3.4 | 3.6 | 3.5 | 3.4 | 4.1 | 2.9 |
| ORN^*^ | Y=1256.82X+597.082 | 0.993076 | 1~150 | 3.2 | 5.3 | 3.3 | 5.0 | 3.9 | 3.6 |
| CYS^*^ | Y=3833.52X+1154.99 | 0.991805 | 2~400 | 5.0 | 4.1 | 5.1 | 4.0 | 5.7 | 3.6 |
| LEU^*^ | Y=0.0269792X+0.00766949 | 0.994225 | 2~400 | 3.2 | 5.3 | 3.3 | 5.0 | 3.9 | 4.3 |
| ILE^*^ | y = 0.0237906x+0.0093575 | 0.988483 | 2~400 | 0.8 | 7.9 | 0.9 | 6.7 | 1.5 | 4.1 |
| TRP^*^ | Y=0.135964X+0.0271021 | 0.992640 | 2~400 | 2.9 | 4.9 | 3.1 | 4.7 | 3.7 | 3.9 |
| SAR^*^ | Y=1888.122X+30.273 | 0.994113 | 1~150 | 0.9 | 6.0 | 1.0 | 5.2 | 1.6 | 5.2 |

**(continued)**

**Supplementary Table 4**

**(continued)**

| **Compound** | **Linear equation** | **r^2^** | **Linearity range（μM）** | **QCL** | | **QCM** | | **QCH** | |
| --- | --- | --- | --- | --- | --- | --- | --- | --- | --- |
|  |  |  |  | **RE/%** | **RSD/%** | **RE/%** | **RSD/%** | **RE/%** | **RSD/%** |
| LYS^*^ | Y=0.0779555X+0.0263836 | 0.995462 | 2~400 | 5.7 | 3.4 | 5.9 | 3.3 | 6.5 | 3.0 |
| γ-GABA^&^ | Y=18576.5X+5310.3 | 0.988575 | 0.1~40 | -0.2 | -3.8 | -0.1 | -10.9 | 0.5 | 1.8 |
| CIT^*^ | Y=2733.91X+3753.47 | 0.992447 | 1~200 | 0.9 | 6.0 | 1.0 | 5.2 | 1.6 | 3.3 |
| AMADP^&^ | Y=14125X+3788.39 | 0.991335 | 0.1~40 | 7.3 | 1.3 | 7.4 | 1.3 | 8.0 | 1.2 |
| CAR^*^ | Y=304.688X+104.585 | 0.990383 | 1~200 | 10.3 | 1.6 | 10.4 | 1.6 | 11.0 | 1.5 |
| PRO^*^ | Y=0.00694718X+0.00165202 | 0.994870 | 2~400 | 8.7 | 2.0 | 8.8 | 2.0 | 9.8 | 1.8 |
| TYR^*^ | Y=0.0990658X+0.0255132 | 0.994419 | 2~400 | 12.6 | 1.0 | 12.6 | 1.0 | 13.7 | 1.0 |
| MET^*^ | Y=0.129596X+0.0213 | 0.995034 | 2~200 | 1.6 | 1.3 | 1.7 | 1.3 | 2.7 | 0.8 |
| VAL^*^ | Y=0.0148855X+0.0039265 | 0.993896 | 2~400 | 3.4 | 2.9 | 3.5 | 2.9 | 4.5 | 2.2 |
| PHE^*^ | Y=0.0405157X+0.00841412 | 0.994966 | 2~200 | 8.7 | 2.0 | 8.7 | 2.0 | 9.8 | 1.8 |

* The corresponding QC concentrations of the compounds were 3 μM, 30 μM, and 120 μM, respectively.

^#^ The corresponding QC concentrations of the compounds were 60 μM, 600 μM, and 1200 μM, respectively.

^&^ The corresponding QC concentrations of the compounds were 0.3 μM, 3 μM, and 30 μM, respectively.

**Table S5 Matrix effects for the analytes**

| **Compound** | **QCL** | **QCM** | **QCH** |
| --- | --- | --- | --- |
|  | **matrix effects**  **(%, mean±SD)** | **matrix effects**  **(%, mean±SD)** | **matrix effects**  **(%, mean±SD)** |
| HIS^*^ | 110.51±12.01 | 99.02±9.78 | 102.70±5.08 |
| 1-MEHIS^*^ | 118.55±10.30 | 118.97±5.09 | 116.16±9.41 |
| 3-MEHIS^*^ | 112.53±8.06 | 93.97±8.97 | 88.86±3.75 |
| ASN^*^ | 116.91±9.13 | 112.77±6.03 | 113.72±6.50 |
| ARG^*^ | 99.22±1.27 | 98.46±5.90 | 104.74±3.96 |
| ANS^*^ | 81.85±11.31 | 84.77±4.02 | 84.15±2.26 |
| TAU^#^ | 105.92±7.50 | 100.90±13.65 | 100.92±11.23 |
| GLN^#^ | 90.47±4.16 | 93.63±5.05 | 97.75±2.95 |
| SER^*^ | 113.85±9.87 | 98.51±10.20 | 101.28±13.19 |
| ETH^*^ | 82.58±9.00 | 85.41±3.39 | 81.19±5.59 |
| GLY^#^ | 105.44±8.66 | 106.46±4.66 | 108.48±4.12 |
| ASP^*^ | 81.56±9.05 | 89.64±6.51 | 91.52±5.67 |
| GLU^*^ | 106.82±6.14 | 117.29±11.06 | 107.69±5.70 |
| ALA^*^ | 94.47±5.25 | 97.07±7.44 | 111.34±3.06 |
| THR^*^ | 96.73±5.98 | 84.72±4.66 | 89.14±4.39 |
| β-GABA^*^ | 95.00±11.18 | 98.51±7.32 | 90.78±9.09 |
| OH-LYS^*^ | 109.67±5.72 | 113.53±4.80 | 120.07±2.36 |
| ORN^*^ | 106.87±6.08 | 108.02±8.93 | 115.81±4.87 |
| CYS^*^ | 101.29±6.13 | 105.90±7.42 | 109.89±7.21 |
| LEU^*^ | 92.24±13.50 | 111.49±4.30 | 88.41±5.39 |
| ILE^*^ | 81.26±5.85 | 108.85±2.80 | 87.30±6.40 |
| TRP^*^ | 111.46±4.88 | 102.10±6.85 | 101.58±4.55 |
| SAR^*^ | 114.35±4.31 | 108.68±6.07 | 113.86±4.24 |
| LYS^*^ | 105.85±5.70 | 112.01±6.96 | 111.56±5.76 |
| γ-GABA^&^ | 100.00±11.18 | 101.17±6.55 | 96.99±3.10 |
| CIT^*^ | 117.78±7.14 | 92.68±3.94 | 94.41±5.01 |
| AMADP^&^ | 112.76±12.40 | 100.68±3.41 | 101.81±3.96 |
| CAR^*^ | 111.28±13.08 | 101.34±10.39 | 107.04±8.12 |
| PRO^*^ | 103.23±6.25 | 104.34±8.00 | 103.32±8.00 |
| TYR^*^ | 82.95±16.04 | 94.31±4.47 | 92.07±8.86 |
| MET^*^ | 88.24±12.74 | 91.78±4.53 | 96.73±8.28 |
| VAL^*^ | 91.86±14.89 | 97.82±3.19 | 95.51±6.66 |
| PHE^*^ | 119.14±8.70 | 105.98±4.10 | 111.11±7.27 |

* The corresponding QC concentrations of the compounds were 3 μM, 30 μM, and 120 μM, respectively.

^#^ The corresponding QC concentrations of the compounds were 60 μM, 600 μM, and 1200 μM, respectively.

^&^ The corresponding QC concentrations of the compounds were 0.3 μM, 3 μM, and 30 μM, respectively.

Supplementary Figure 1 The box plots of clinical features with significant digits

Supplementary Figure 2 PCA with QC
